# A new automatic geo-electric self-potential imaging technique for diverse sustainable development scenarios

**DOI:** 10.1038/s41598-024-54689-3

**Published:** 2024-03-14

**Authors:** Mahmoud Elhussein, Zein E. Diab

**Affiliations:** Department of Geophysics, Faculty of Science, Cairo University, P.O. 12613, Giza, Egypt

**Keywords:** Self-potential, Imaging, Local wavenumber, Geothermal exploration, Mineral exploration, Solid Earth sciences, Geodynamics, Geology, Geophysics, Mineralogy

## Abstract

This study introduces a rapid and efficient inversion algorithm designed for the interpretation of self-potential responses originating from mineralized and ore sources and hydrothermal activity, specifically addressing spherical, vertical, and horizontal cylindrical structures. The algorithm leverages local wavenumber and correlation imaging techniques to enhance accuracy in modeling. The correlation factor (*C*_*f*_ value) is crucial in this approach, calculated as the correlation between the local wavenumber of the measured self-potential field and that of the computed field. The algorithm identifies the maximum correlation *C*_*f*_ value (C_**F**_-max) as indicative of the optimal true model parameters. To validate the proposed algorithm, it was applied to three theoretical examples—one with contamination from regional background and another with multiple sources with and without different types of noises (random Gaussian and white Gaussian noises). Additionally, the approach was tested on three distinct real field cases related to mining, ore investigation and hydrothermal activity in India, Germany and USA. Through a comprehensive analysis of results from theoretical and real-world scenarios, including comparisons with different available data and literature information, the study concludes that the method is effective, applicable to multiple sources, accurate, and does not necessitate prior knowledge of the source shape. This algorithm presents a promising advancement in the field of self-potential interpretation for mineral exploration and geothermal exploration.

## Introduction

The self-potential (SP) or spontaneous polarization method stands out as an exceptionally passive approach within geophysics^1–3^. It gauges the inherent potential difference (∆V) beneath the surface, resulting from electrochemical, thermoelectric, and electrokinetic fields present within the Earth's interior^4–7^. A myriad of geophysical challenges can be effectively addressed using the self-potential technique, encompassing tasks like delineating paleo-shear zones, mining, groundwater exploration, archaeology, geothermal investigation, and identifying underground voids^8–11^.

Given the challenges arising from non-unique and ill-posed situations when interpreting self-potential anomalies linked to diverse mineralized sources, various inversion modeling methods have been devised to tackle these issues^5,12,13^. These inversion techniques primarily entail approximating the various geoelectric sources through uncomplicated geometric configurations to deduce structural parameters^6,14–17^. These methodologies encompass a range of strategies such as the linear and non-linear inversion approach^18–20^, utilization of nomograms and the graphical approach^21–24^, neural networks^25^, and gradient approach^11,26^. Many of these methods necessitate prior knowledge about the model's parameters and a suitable parameter search range to find optimal solutions. Pateela^27^ introduced SP tomography^28,29^, a technique that entails scanning a segment within an SP survey profile utilizing a basic charge unit of uniform strength. This charge is administered across a systematic grid of spatial coordinates, and the probability function for charge occurrence is computed at each individual point. The resulting set of grid values enables the creation of contour lines, aiding in identifying areas with the highest probability of concentrated polarized, primary, and secondary electric charges. However, utilizing this approach to calculate the depth of a source necessitates access to the structural index of the causative source, a task that proves challenging in the case of an unfamiliar region^30^.

The more recent advancements include techniques like genetic algorithms^31,32^, black hole technique^33^; particle swarm optimization (PSO)^6,34^, grey wolf optimization^35^, simulated and very fast simulated annealing^31,36^. These methods provide a primary benefit by efficiently exploring extensive solution spaces without requiring prior familiarity with the underlying structure of the problem. However, some of these methods face challenges when dealing with multi-structure optimization. Additionally, others may face issues in achieving enhanced search output, necessitating tuning of parameters. An enhanced control strategy is required to adeptly transition between exploration and exploitation^37^. While these techniques do not ensure the discovery of the optimal outcome, their objective is to identify optimal solutions within a reasonable timeframe^38^.

This study has introduced an effective imaging algorithm that has been devised to comprehensively interpret self-potential data stemming from diverse subterranean structures like horizontal cylinders, spheres, and vertical cylinders. This method hinges on the computation of the correlation factor (*C*_*f*_) between the local wavenumber of the observed self-potential anomaly and that of the calculated anomaly. The model associated with the highest *C*_*f*_ value (C_**F**_-max) is deemed the most accurate model. This strategy has potential applications in diverse fields such as mineral and ore exploration, as it aids in determining various structural parameters including amplitude factor (*K*), depth (*z*_*o*_), body origin (*x*_*o*_), shape factor (*q*), and polarization angle ($\theta )$ all without requiring any prior knowledge of the source shape. Furthermore, this technique can also be extended to estimating parameters from multiple sources. In order to validate the effectiveness and practicality of this proposed approach, the method was employed to analyze self-potential data from three theoretical scenarios with and without different types of noises, as well as three field examples from India, Germany and USA.

## Methodology

**Figure 1:**
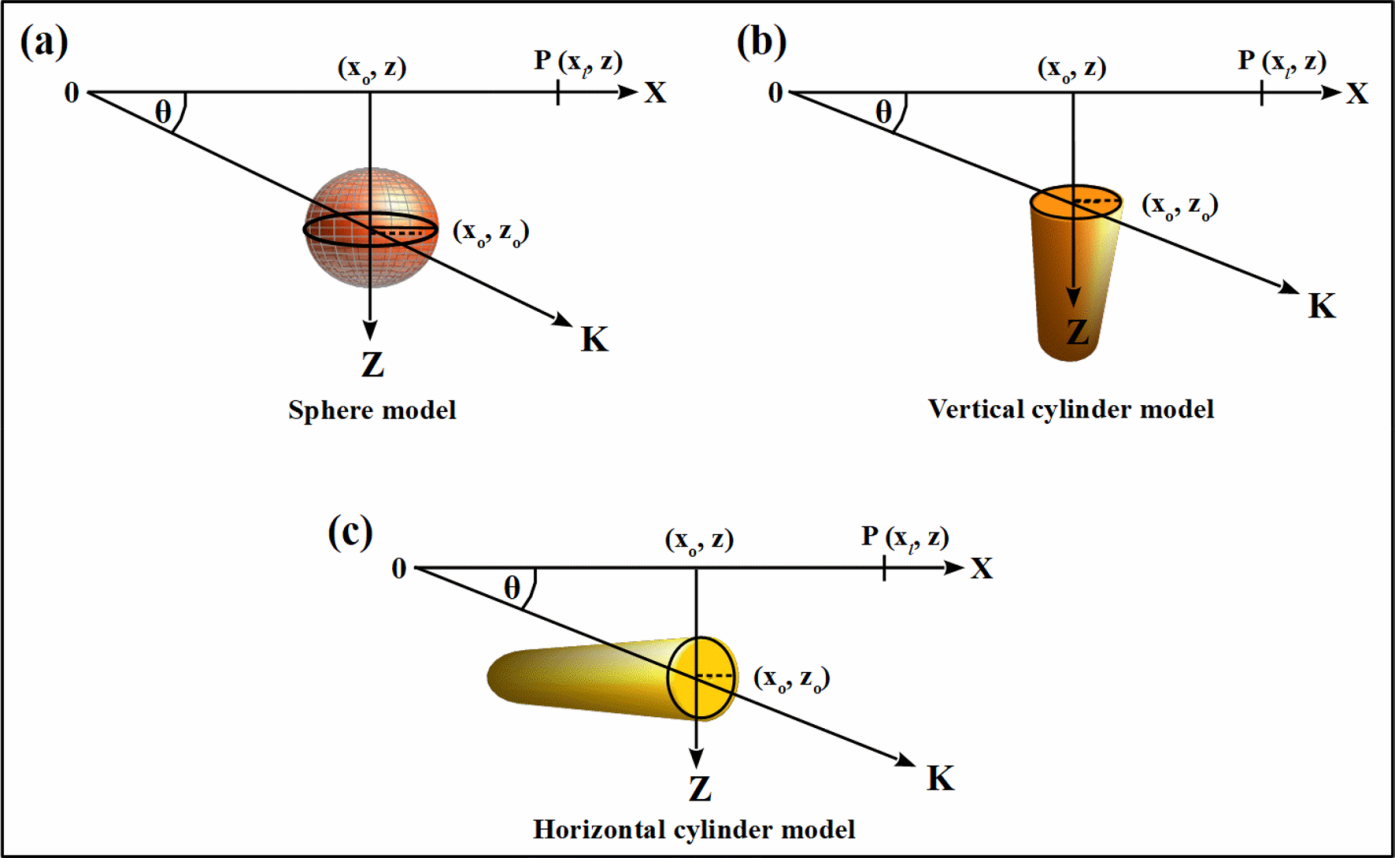
Different geometric structures for various basic shapes include (**a**) sphere, (**b**) vertical cylinder, (**c**) horizontal cylinder.

The self-potential signature (P) at an observation point (x_j_, z) along the profile depicted in Fig. 1, can be expressed using the formula of Yüngül^39^.

1 $$P\left(K,{x}_{j},{x}_{o},{z}_{o},\theta , q\right)=K\frac{\left({x}_{l}-{x}_{o}\right){\text{cos}}\theta +{z}_{o} sin \theta }{{\left[{ \left({x}_{l}-{x}_{o}\right)}^{2}+{{z}_{o}}^{2} \right]}^{q}}, j=\mathrm{1,2},3,\dots , n$$

where *n* represents the count of data points, *q* is the shape factor, a dimensionless quantity, varies according to the structure's shape (it takes a value of 1.5 for a structure resembling a sphere, 1 for a horizontal cylindrical structure, and 0.5 for a semi-infinite vertical cylinder. The depth of the structure is denoted as '*z*_*o*_' in meters. The amplitude factor (*K*), with unit $\mathrm{mV }{{\text{m}}}^{2q-1}$, the parameter '*x*_*o*_' indicates the position of the source body in meters, and '$\theta$' corresponds to the polarization angle in degrees.

The measured local wavenumber can be formulated by^30,40^:

2 $${LW}_{mea}=\frac{\partial \varnothing }{\partial x},$$

where

3 $$\varnothing ={{\text{tan}}}^{-1}\left[\frac{\left(\frac{\partial P}{\partial z}\right)}{\left(\frac{\partial P}{\partial x}\right)}\right],$$

By substitution (Eq. 3 in Eq. 2), ${LW}_{mea}$ can be given by:

4 $${LW}_{mea}=\frac{\partial }{\partial x} {{\text{tan}}}^{-1}\left[\frac{\left(\frac{\partial P}{\partial z}\right)}{\left(\frac{\partial P}{\partial x}\right)}\right]=\frac{1}{{AS}^{2}} \left[\left(\frac{{\partial }^{2}P}{\partial x\partial z}.\frac{\partial P}{\partial x}\right)-\left(\frac{{\partial }^{2}P}{\partial {x}^{2}}.\frac{\partial P}{\partial z}\right)\right],$$

where *AS* is the analytical signal amplitude as follow^41^:

5 $$AS=\sqrt{{\left(\frac{\partial P}{\partial x}\right)}^{2}+{\left(\frac{\partial P}{\partial z}\right)}^{2}},$$

By applying the horizontal and vertical derivatives $$\left(\frac{\partial P}{\partial x}\right)$$ and $$\left(\frac{\partial P}{\partial z}\right)$$ respectively to Eq. (1) and substituting in Eq. (4), the computed local wavenumber ($${LW}_{com})$$ is given by:

6 $${LW}_{com}=\frac{-2q{z}_{o}\left[\left({({x}_{l}-{x}_{o})}^{2}+{{z}_{o}}^{2}\right)\left(\frac{q}{q-1}-{\text{cos}}2\theta \right)+2{z}_{o}\left({x}_{l}-{x}_{o}\right){\text{sin}}2\theta \right]}{\left({({x}_{l}-{x}_{o})}^{2}+{{z}_{o}}^{2}\right)\left[\frac{1}{q-1}+2q\left(\left({({x}_{l}-{x}_{o})}^{2}-{{z}_{o}}^{2}\right){\text{cos}}2\theta +2{z}_{o}\left({x}_{l}-{x}_{o}\right){\text{sin}}2\theta +1\right)\right]},$$

Using ${LW}_{mea}$ and ${LW}_{com}$, the correlation parameter can be represented by^30,40^:

7 $${C}_{F}=\frac{{\sum }_{j=1}^{n}|{LW}_{mea}{|}_{j}|{LW}_{com}{|}_{j}}{\sqrt{{\sum }_{j=1}^{n}|{LW}_{mea}{{|}_{j}}^{2}{\sum }_{j=1}^{n}|{LW}_{com}{{|}_{j}}^{2}}}.$$

**Figure 2:**
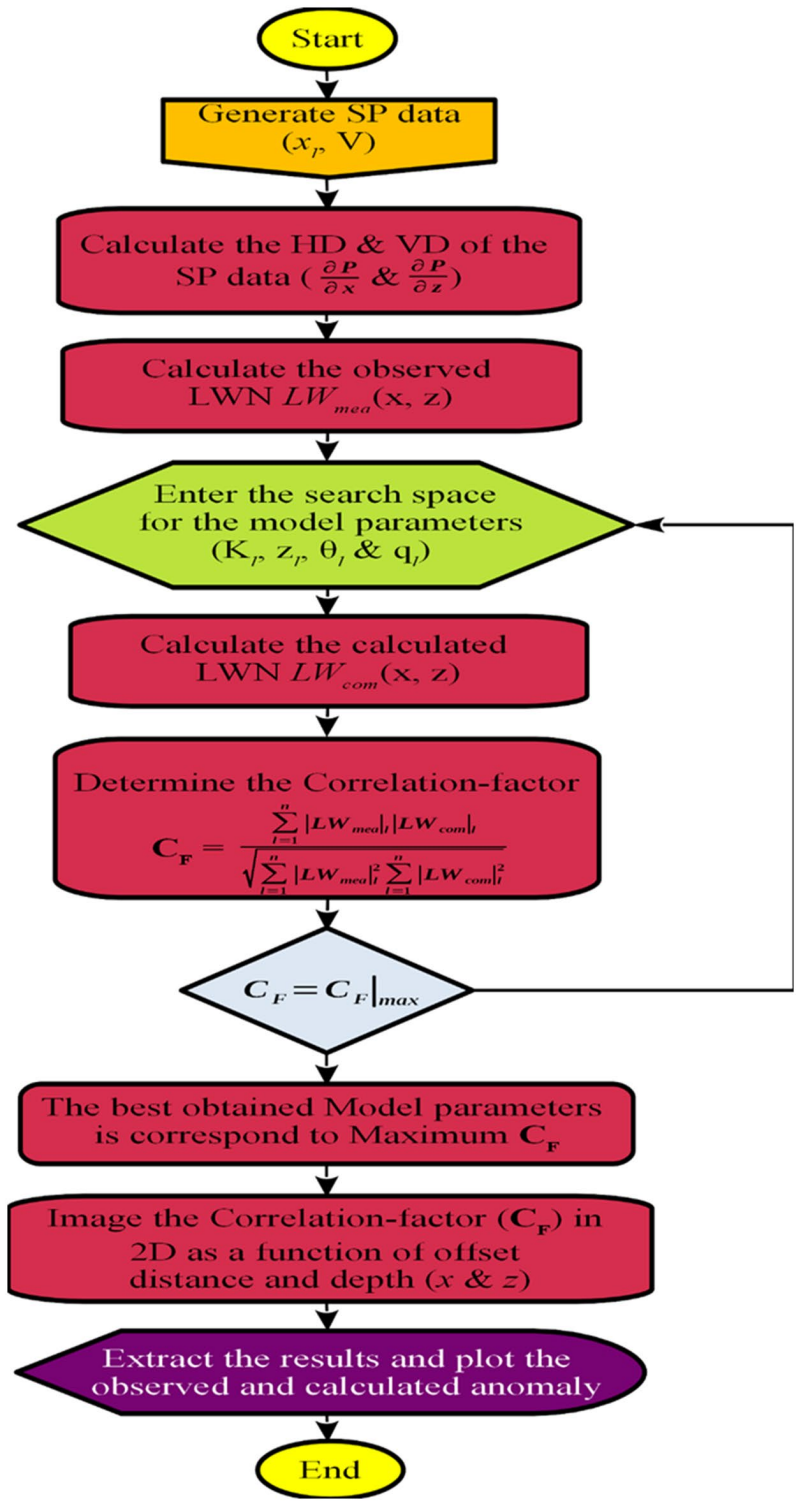
Flowchart depicting the procedural sequence of the algorithm under consideration.

Using Eq. (7), the calculation of the correlation parameter (${C}_{F}$) between ${LW}_{mea}$ and ${LW}_{com}$ is performed, and the highest value of ${C}_{F}$ corresponds to actual body characteristics^30,40^. The process flow of the proposed algorithm is illustrated in Fig. 2. After identifying the most suitable parameters from the search space based on the highest ${C}_{F}$ value (C_F_-max), it becomes possible to create a two-dimensional representation of ${C}_{F}$ for the preferred source (specifically, the shape factor *q*) in relation to subsurface depth (m). The solid black dot present in the imaging section symbolizes the accurate position for both depth and location.

## Synthetic models

This segment demonstrates the application of the suggested method to three distinct synthetic models, both with and without noise, in order to assess the effectiveness and suitability of the proposed approach in the interpretation of self-potential anomalies.

### Example 1

**Figure 3:**
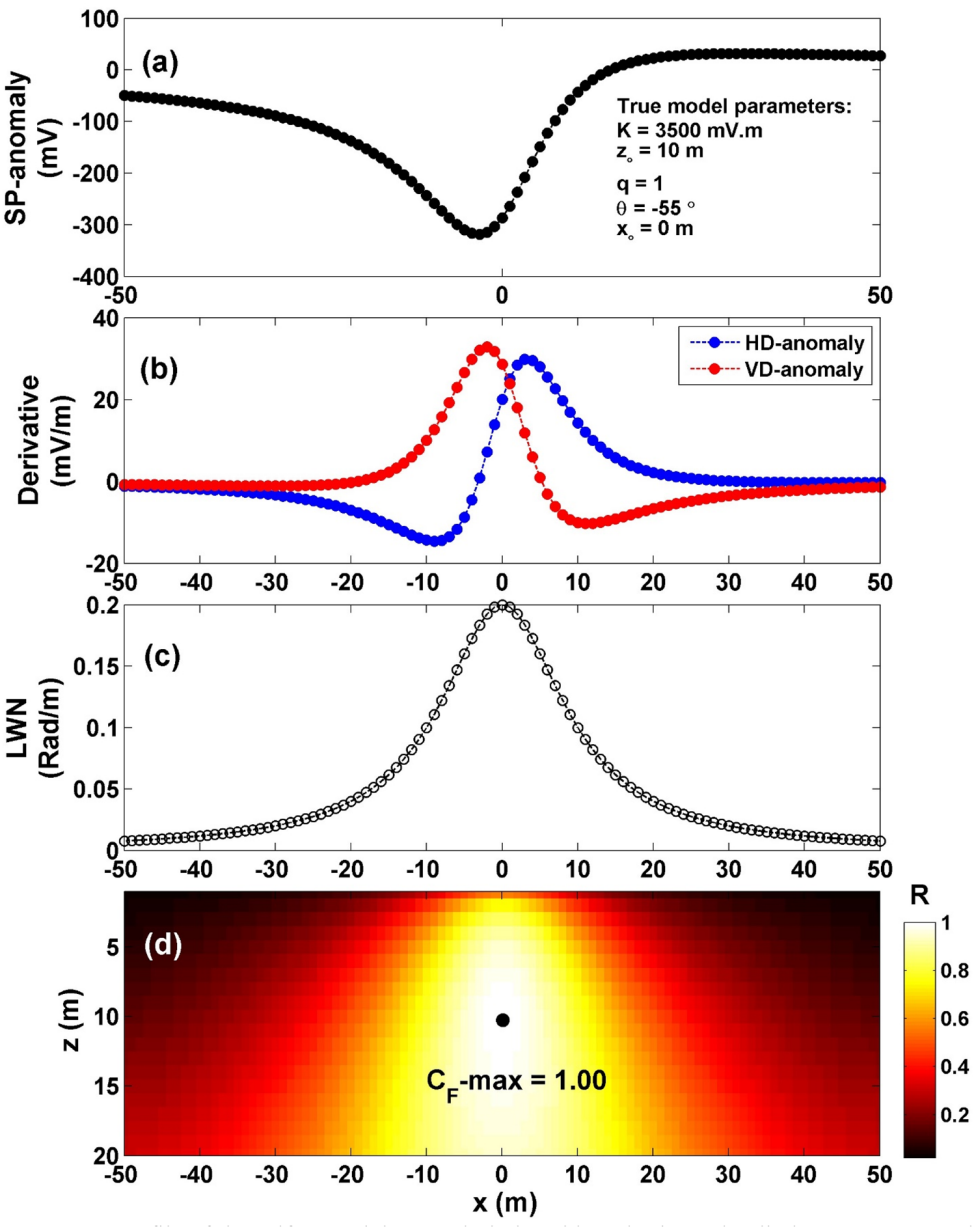
(**a**) Profile of the self-potential anomaly induced by a horizontal cylinder, (**b**) computed horizontal and vertical derivatives for the profile depicted in (**a**), (**c**) local wavenumber of the data depicted in (**b**), (**d**) visualizing the correlation factor (*C*_*f*_) and determining the C_**F**_-max through the newly established method.

The self-potential profile resulting from a horizontal cylinder was computed with specific parameters: *K* = 3500 mV m, *z*_*o*_ = 10 m, *x*_*o*_ = 0 m, *q* = 1, and *θ* = -55°, over a profile length of 100 m (depicted in Fig. 3a). The interpretation process began by calculating both horizontal and vertical gradients of the observed anomaly (as shown in Fig. 3b). Subsequently, the value of ${LW}_{mea}$ was determined using Eq. (4) (illustrated in Fig. 3c). Moving forward, the calculation of *C*_*f*_ was carried out using Eq. (7) (as demonstrated in Fig. 3d), considering various *q* values as presented in Table 1. Notably, in Table 2, the highest value of *C*_*f*_ (C_**F**_-max = 1) (black circle in Fig. 3d) is located at *K* = 3500 mV m, *z*_*o*_ = 10 m, *x*_*o*_ = 0 m,* q* = 1, and *θ* = -55°, which aligns with the information in Fig. 3d. This outcome signifies the exceptional efficiency of the proposed method. Utilizing the suggested approach facilitated the estimation of inverted parameters as detailed in Table 2, leading to a complete absence of errors for the diverse parameters.

**Table 1:** The correlation factor (**C**_**F**_) calculated at the different shape factors for the first theoretical example (self-potential anomaly induced by a horizontal cylinder).

Geometric shape factor	Maximum correlation factor
**(q)**	**(C**_**F**_**-max)**
0.5	0.9242
**1**	**1.0000**
1.5	0.9995

The optimum values are in [bold].

**Table 2:** The authentic and retrieved model parameters pertaining to the first theoretical example (self-potential anomaly generated by a horizontally cylinder).

Model parameters	True	Recovered
K (mV m)	3500	3500
*z*_*o*_ (m)	10	10
*x*_*o*_ (m)	0	0
q	1.0	1.0
θ (°)	− 55	− 55
C_F_-max		1.0000

**Figure 4:**
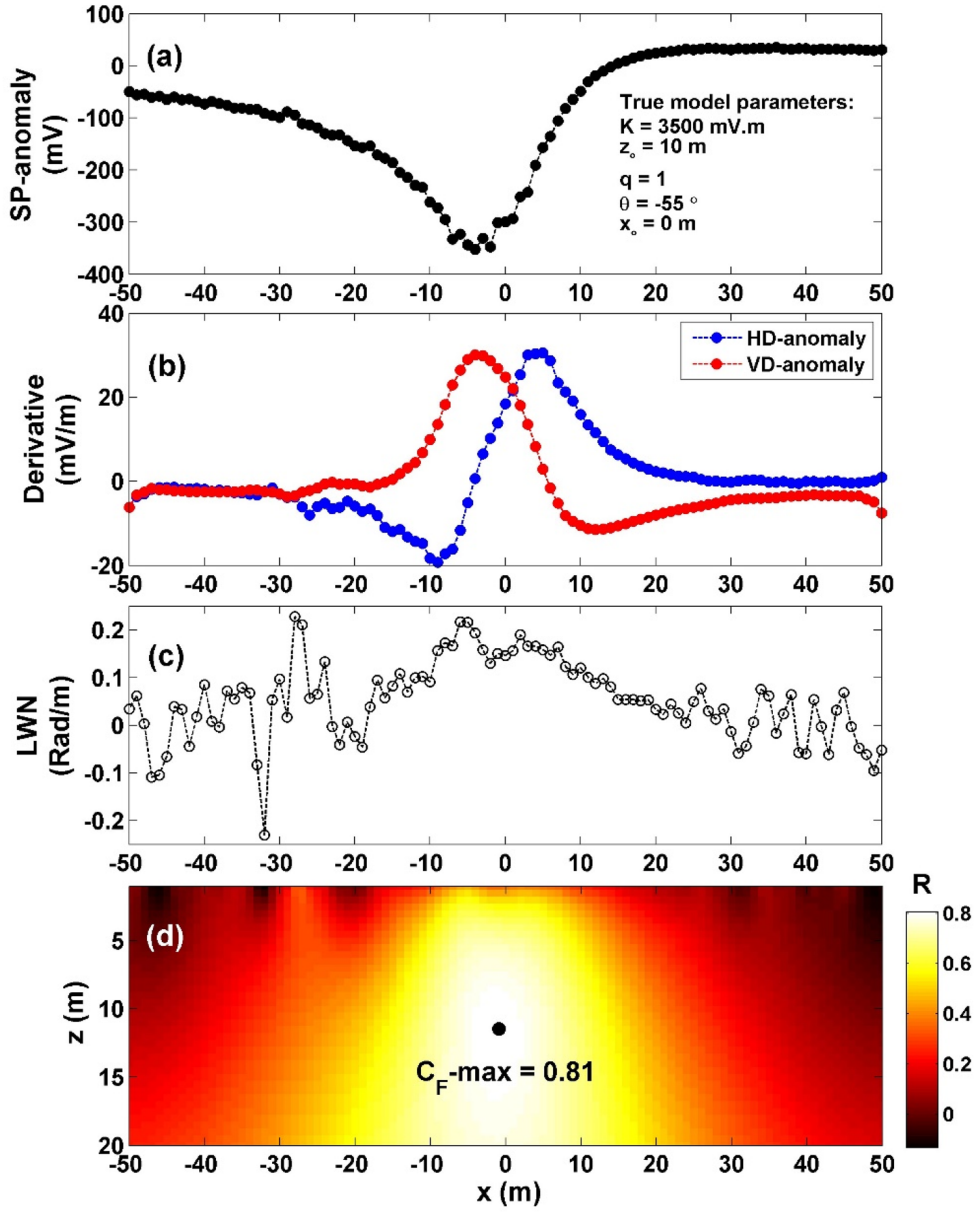
(**a**) Profile of the self-potential anomaly depicted in Fig. 3a after contaminating with 15% RGN, (**b**) computed horizontal and vertical derivatives for the profile depicted in (**a**), (**c**) local wavenumber of the data depicted in (**b**), (**d**) visualizing the correlation factor (*C*_*f*_) and determining the C_**F**_-max through the newly established method.

To assess the robustness and effectiveness of the suggested method when applied to data with noise, the method was applied to the previous model after adding 15% random Gaussian noise (RGN) and 15% white Gaussian noise (WGN). Firstly, 15% RGN (Fig. 4a), the noisy data's vertical and horizontal gradients were computed (Fig. 4b). Subsequently, utilizing Eq. (4), ${LW}_{mea}$ was determined (Fig. 4c). To derive *C*_*f*_, Eq. (7) was employed (Fig. 4d). Within Fig. 4d, the highest *C*_*f*_ value of 0.8075 (depicted by the black circle in Fig. 4d) is observed at *K* = 3948 mV m, *z*_*o*_ = 11.5 m, *x*_*o*_ = − 1 m,* q* = 1, and *θ* = − 54°, as indicated in Table 3. The computed error of the estimated parameters, *K*, *z*_*o*_,* q*, *θ* are: 12.8%, 15%, 0% and 1.82% respectively.

**Table 3:** The authentic and retrieved model parameters pertaining to the first theoretical example (self-potential anomaly generated by a horizontally cylinder) contaminated with 15% RGN and 15% WGN.

Model parameters	True	Recovered	
		15% RGN	15% WGN
K (mV m)	3500	3948	4015
*z*_*o*_ (m)	10	11.5	12
*x*_*o*_ (m)	0	− 1	− 1
q	1.0	1.0	1.0
θ (º)	− 55	− 54	− 54.5
C_F_-max		0.8075	0.6784

**Figure 5:**
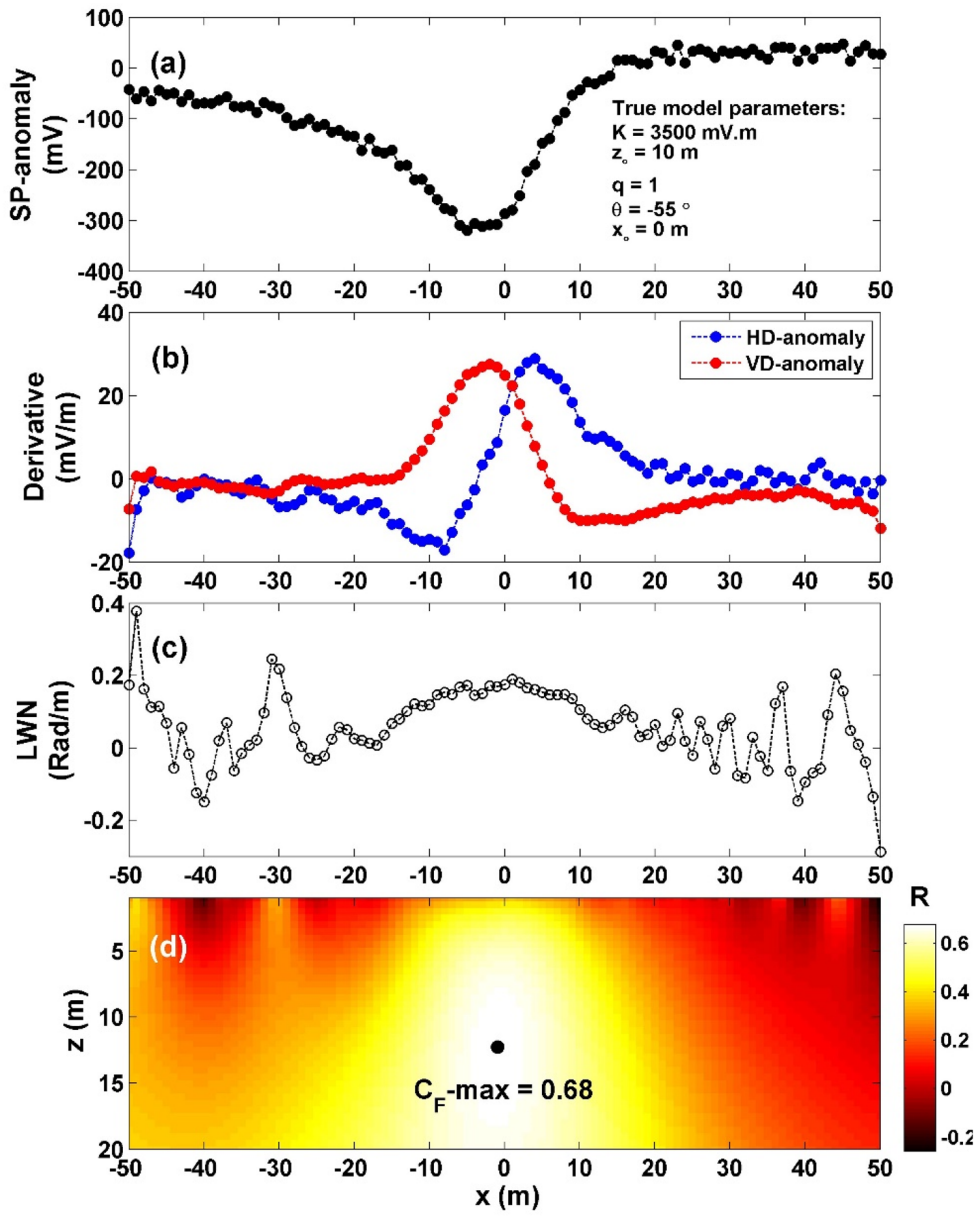
(**a**) Profile of the self-potential anomaly depicted in Fig. 3a after contaminating with 15% WGN, (**b**) computed horizontal and vertical derivatives for the profile depicted in (**a**), (**c**) local wavenumber of the data depicted in (**b**), (**d**) visualizing the correlation factor (*C*_*f*_) and determining the C_**F**_-max through the newly established method.

Secondly, 15% WGN (Fig. 5a), the noisy data's vertical and horizontal gradients were computed (Fig. 5b). Subsequently, applying Eq. (4), ${LW}_{mea}$ was determined (Fig. 5c). To derive *C*_*f*_, Eq. (7) was employed (Fig. 5d). Within Fig. 5d, the highest *C*_*f*_ value of 0.6784 (depicted by the black circle in Fig. 5d) is observed at *K* = 4015 mV m, *z*_*o*_ = 12 m, *x*_*o*_ = -− 1 m,* q* = 1, and *θ* = − 54.5°, as indicated in Table 3. The computed error of the estimated parameters, *K*, *z*_*o*_,* q*, *θ* are: 14.7%, 20%, 0% and 0.91% respectively. The results obtained above shows that the effect of the WGN is greater than RGN on the proposed method but the estimated parameters in case of the different two types of noise demonstrating that the proposed method can effectively be employed to handle noisy data with exceptional performance.

### Example 2

**Figure 6:**
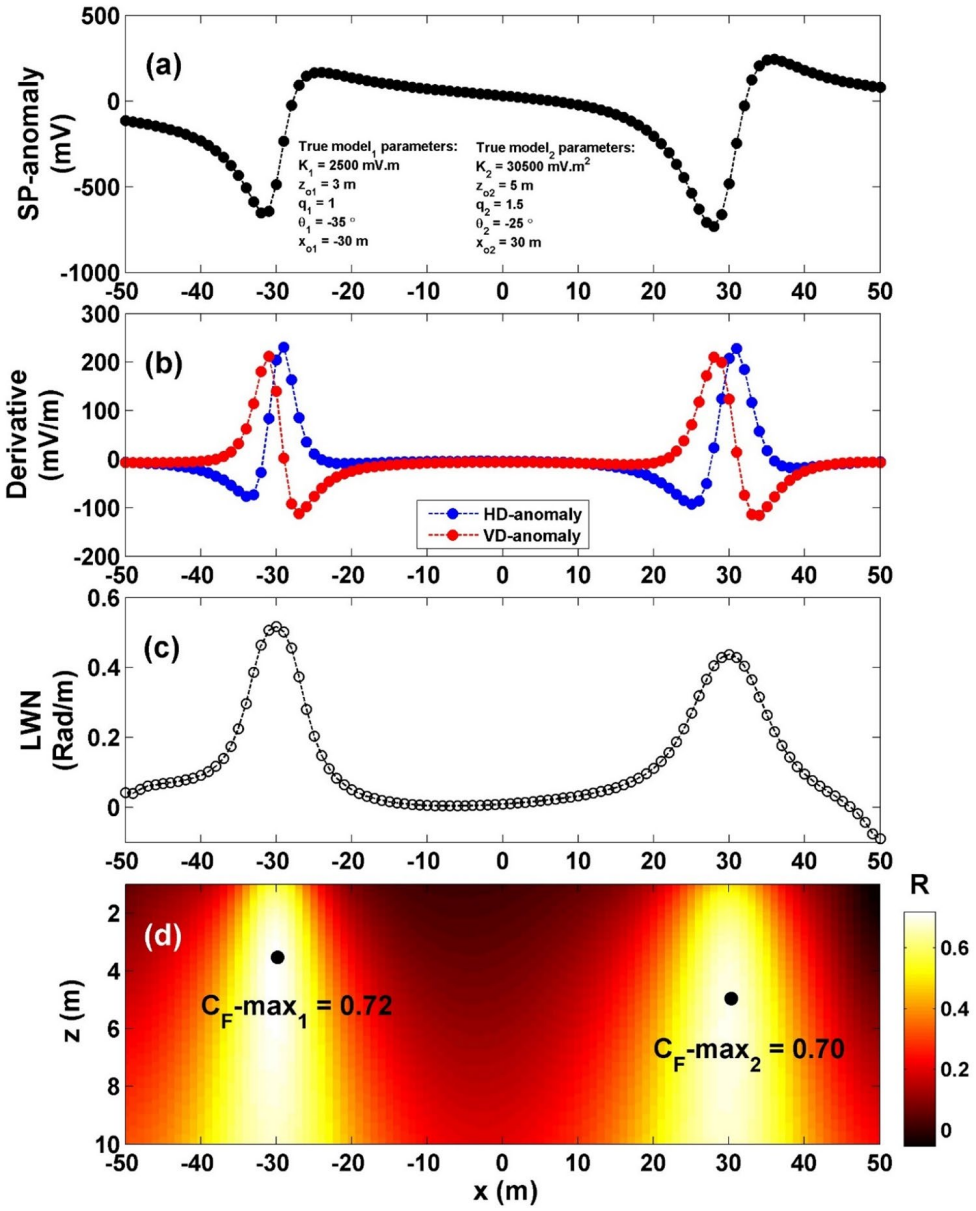
(**a**) Profile of the self-potential anomaly induced by multisource models of sphere and horizontal cylinder, (**b**) computed horizontal and vertical derivatives for the profile depicted in (**a**), (**c**) local wavenumber of the data depicted in (**b**), (**c**) visualizing the correlation factor (*C*_*f*_) and determining the C_**F**_-max through the newly established method.

In order to evaluate the suitability and effectiveness of the employed approach when dealing with multisource examples, the technique was implemented on a 100 m composite profile that was constructed of sphere body (applying these parameters: *K* = 30,500 mV m^2^, *z*_*o*_ = 5 m, *x*_*o*_ = − 30 m,* q* = 1.5, and *θ* = − 25°) and horizontal-cylinder (HC) body (applying these specific parameters: *K* = 2500 mV m, *z*_*o*_ = 3 m, *x*_*o*_ = 30 m,* q* = 1, and *θ* = − 35°) (Fig. 6a). The process of interpretation began with the computation of both the vertical and horizontal gradients of the composite profile (depicted in Fig. 6b). The value ${LW}_{mea}$ was determined applying Eq. (4) (illustrated in Fig. 6c), while the value of *C*_*f*_ was calculated using Eq. (7) (as shown in Fig. 6d). It is demonstrated that the highest *C*_*f*_ value (C_**F**_-max_1_) for the sphere body is 0.74 (indicated by the initial black circle in Fig. 6d), situated at *K* = 27,718 mV m^2^, *z*_*o*_ = 4.9 m, *x*_*o*_ = − 30 m,* q* = 1.5, and *θ* = − 24.71° (Table 4). Similarly, the maximum *C*_*f*_ value (C_**F**_-max_2_) for the HC body is 0.70 (depicted by the second black circle in Fig. 6d), located at *K* = 2636 mV m, *z*_*o*_ = 3.4 m, *x*_*o*_ = 30 m,* q* = 1, and *θ* = − 38.79°, as summarized in Table 4. The computed error of the estimated parameters, *K*, *z*_*o*_,* x*_*o*_,* q*, and *θ* are: 9.12%, 2%, 0%, 0% and 1.16% respectively for the sphere body, while for the HC source, the computed error of the estimated parameters, *K*, *z*_*o*_,* x*_*o*_,* q*, and *θ* are: 5.44%, 13.33%, 0%, 0% and 10.83%, respectively.

**Table 4:** The authentic and retrieved model parameters pertaining to the second theoretical example (induced by multisource models of sphere and horizontal cylinder).

Model parameters	True		Recovered	
	HC model	Sphere model	HC model	Sphere model
K (mV m^2q−1^)	2500 mV m	30,500 mV m^2^	2636 mV m	27,718 mV m^2^
*z*_*o*_ (m)	3	5	3.4	4.9
*x*_*o*_ (m)	30	− 30	30	− 30
q	1.0	1.5	1.0	1.5
θ (º)	− 35	− 25	− 38.79	− 24.71
C_F_-max			0.72	0.70

**Figure 7:**
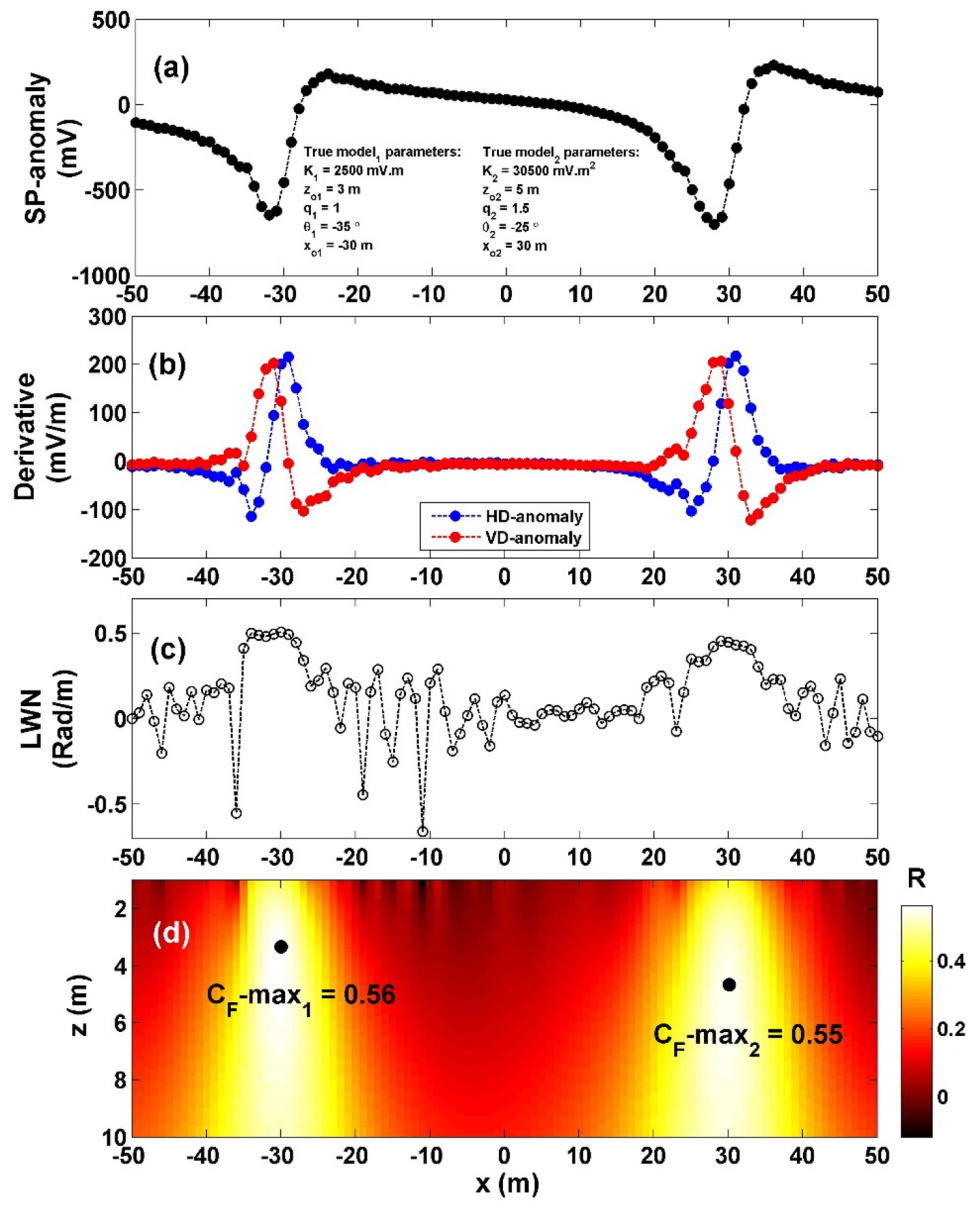
(**a**) Profile of the self-potential anomaly depicted in Fig. 6a after contaminating with 10% RGN, (**b**) computed horizontal and vertical derivatives for the profile depicted in (**a**), (**c**) local wavenumber of the data depicted in (**b**), (**d**) visualizing the correlation factor (*C*_*f*_) and determining the C_**F**_-max through the newly established method.

To evaluate how well the proposed method performs in the presence of noise and its overall effectiveness, the method was applied to the previous model after adding 10% RGN and 10% WGN. The first case, 10% RGN (Fig. 7a), the noisy composite data's vertical and horizontal gradients were computed (Fig. 7b), The value ${LW}_{mea}$ was determined (illustrated in Fig. 7c), while the value of *C*_*f*_ was calculated (as shown in Fig. 7d). It is demonstrated that the highest *C*_*f*_ value (C_**F**_-max_1_) for the sphere body is 0.55 (indicated by the initial black circle in Fig. 7d), situated at *K* = 23,287 mV m^2^, *z*_*o*_ = 4.6 m, *x*_*o*_ = − 30 m,* q* = 1.5, and *θ* = − 26.01° (Table 5). Similarly, the maximum *C*_*f*_ value (C_**F**_-max_2_) for the HC body is 0.56 (depicted by the second black circle in Fig. 7d), located at *K* = 2293 mV m, *z*_*o*_ = 3.2 m, *x*_*o*_ = 30 m,* q* = 1, and *θ* = − 42.67°, as summarized in Table 5. The computed error of the estimated parameters, *K*, *z*_*o*_,* x*_*o*_,* q*, and *θ* are: 23.65%, 8%, 0%, 0% and 4.04% respectively for the sphere body, while for the HC source, the computed error of the estimated parameters, *K*, *z*_*o*_,* x*_*o*_,* q*, and *θ* are: 8.28%, 6.67%, 0%, 0% and 21.91%, respectively.

**Table 5:** The authentic and retrieved model parameters pertaining to the second theoretical example (induced by multisource models of sphere and horizontal cylinder) contaminated with 10% RGN and 10% WGN.

Model parameters	True		Recovered [RGN 10%]		Recovered [WGN 10%]	
	HC model	Sphere model	HC model	Sphere model	HC model	Sphere model
K (mV m^2*q*−1^)	2500 mV m	30,500 mV m^2^	2293 mV m	23,287 mV m^2^	2833 mV m	36,322 mV m^2^
*z*_*o*_ (m)	3	5	3.2	4.6	4.3	5.9
*x*_*o*_ (m)	30	− 30	30	− 30	30	− 30
Q	1.0	1.5	1.0	1.5	1.0	1.5
θ (º)	− 35	− 25	− 42.67	− 26.01	− 47.50	− 27.55
C_F_-max			0.56	0.55	0.48	0.50

**Figure 8:**
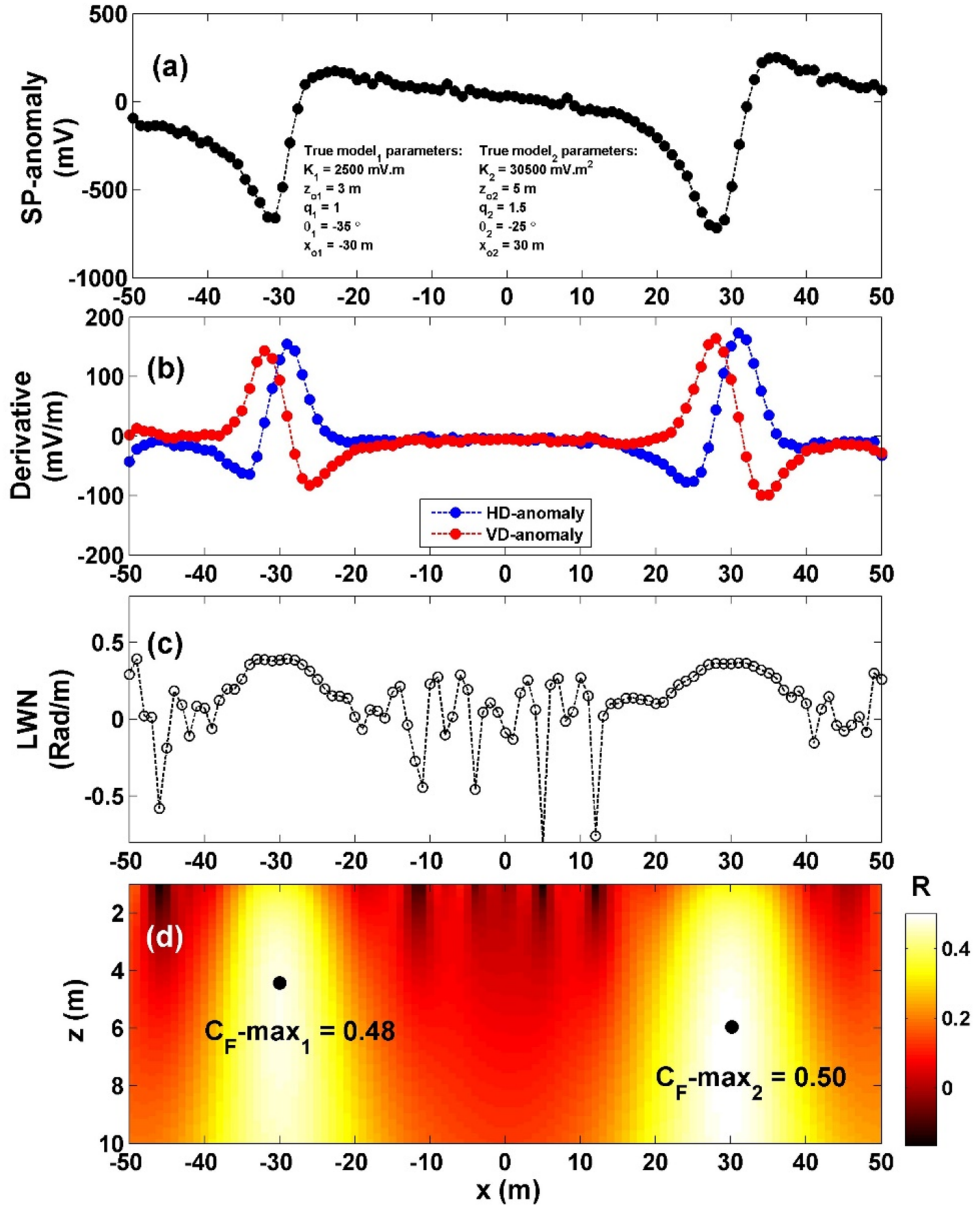
(**a**) Profile of the self-potential anomaly depicted in Fig. 6a after contaminating with 10% WGN, (**b**) computed horizontal and vertical derivatives for the profile depicted in (**a**), (**c**) local wavenumber of the data depicted in (**b**), (**d**) visualizing the correlation factor (*C*_*f*_) and determining the C_**F**_-max through the newly established method.

The second case, 10% WGN (Fig. 8a), the noisy composite data's vertical and horizontal gradients were computed (Fig. 8b), The value ${LW}_{mea}$ was determined (illustrated in Fig. 8c), while the value of *C*_*f*_ was calculated (as shown in Fig. 8d). It is demonstrated that the highest *C*_*f*_ value (C_**F**_-max_1_) for the sphere body is 0.50 (indicated by the initial black circle in Fig. 8d), situated at *K* = 36,322 mV m^2^, *z*_*o*_ = 5.9 m, *x*_*o*_ = − 30 m,* q* = 1.5, and *θ* = − 27.55° (Table 5). Similarly, the maximum *C*_*f*_ value (C_**F**_-max_2_) for the HC body is 0.48 (depicted by the second black circle in Fig. 8d), located at *K* = 2833 mV m, *z*_*o*_ = 4.3 m, *x*_*o*_ = 30 m,* q* = 1, and *θ* = − 47.50°, as summarized in Table 5. The computed error of the estimated parameters, *K*, *z*_*o*_,* x*_*o*_,* q*, and *θ* are: 19.09%, 18%, 0%, 0% and 10.2% respectively for the sphere body, while for the HC source, the computed error of the estimated parameters, *K*, *z*_*o*_,* x*_*o*_,* q*, and *θ* are: 13.32%, 43.33%, 0%, 0% and 35.71%, respectively.

Hence, it can be inferred that the suggested approach is well-suited for scenarios involving multiple sources.

### Example 3

**Figure 9:**
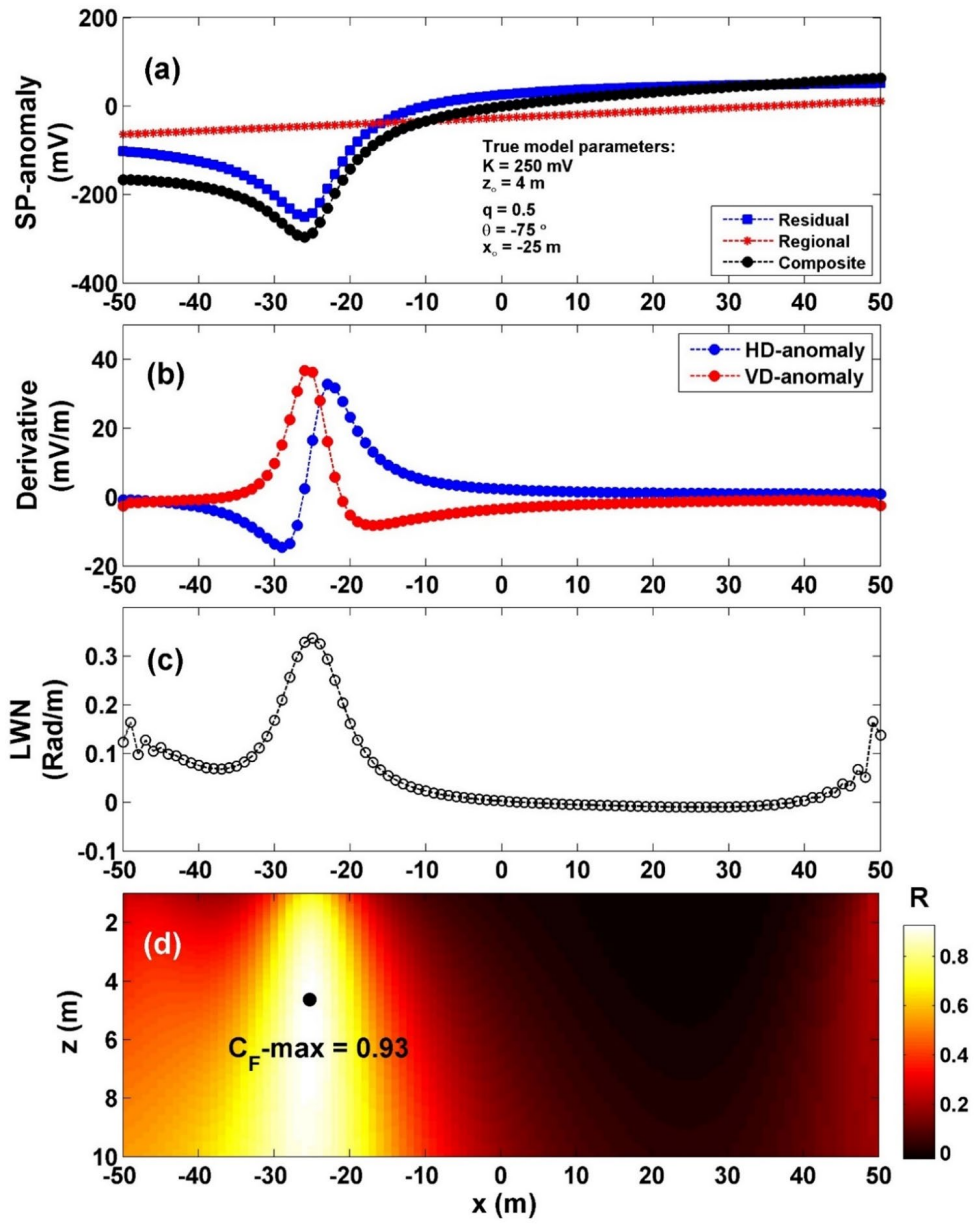
(**a**) Profile of the self-potential composite anomaly induced by vertical cylinder and first order regional source, (**b**) computed horizontal and vertical derivatives for the profile depicted in (**a**), (**c**) local wavenumber of the data depicted in (**b**), (**d**) visualizing the correlation factor (*C*_*f*_) and determining the C_**F**_-max through the newly established method.

To assess the effectiveness of our approach when dealing with a regional context, we introduced a self-potential anomaly profile originating from a vertical cylinder (characterized by: *K* = 250 mV, *z*_*o*_ = 4 m, *x*_*o*_ = − 25 m,* q* = 0.5, and *θ* = − 75^o^ and profile length 100 m) into a deep-seated first order regional anomaly (Fig. 9a). The interpretation process commenced by computing both the horizontal and vertical gradients of the observed anomaly, as depicted in Fig. 9b. Subsequently, Eq. (4), was employed to ascertain the value of ${LW}_{mea}$ (Fig. 9c). Moving forward, Eq. (7) was utilized, to calculate *C*_*f*_ (Fig. 9d). It's worth noting that in Table 6, the highest value of *C*_*f*_ (C_**F**_-max = 0.93), denoted by a black circle in Fig. 9d, corresponds to *K* = 296.58 mV, *z*_*o*_ = 4.5 m, *x*_*o*_ = − 25 m,* q* = 0.5, and *θ* = − 75°. The computed error of the estimated parameters, *K*, *z*_*o*_,* x*_*o*_,* q*, and *θ* are: 18.63%, 12.5%, 0%, 0% and 0%, respectively.

**Table 6:** The authentic and retrieved model parameters pertaining to the second theoretical example (self-potential composite anomaly induced by vertical cylinder and first order regional source).

Model parameters	True	Recovered
K (mV)	250	296.58
*z*_*o*_ (m)	4	4.5
*x*_*o*_ (m)	− 25	− 25
q	0.5	0.5
θ (º)	− 75	− 75
C_F_-max		0.9266

To evaluate the robustness and efficacy of the proposed method when applied to noisy data, we introduced two types of noise, specifically, 15% RGN and 15% WGN, to the previous model.

**Figure 10:**
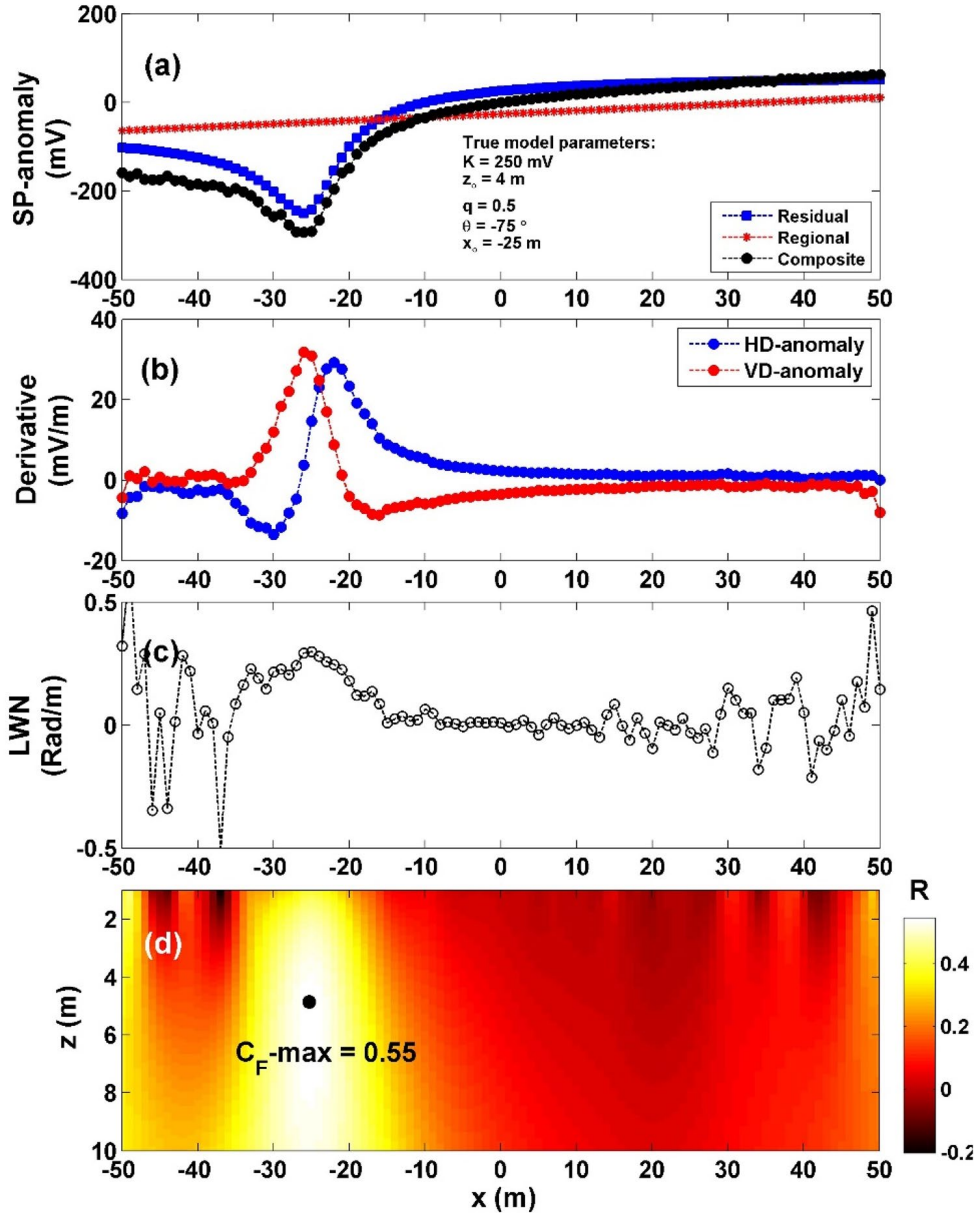
(**a**) Profile of the self-potential anomaly depicted in Fig. 9a after contaminating with 15% RGN, (**b**) computed horizontal and vertical derivatives for the profile depicted in (**a**), (**c**) local wavenumber of the data depicted in (**b**), (**d**) visualizing the correlation factor (*C*_*f*_) and determining the C_**F**_-max through the newly established method.

In the case of 15% RGN (depicted in Fig. 10a), we initially computed the vertical and horizontal gradients of the noisy data (Fig. 10b). Subsequently, using Eq. (4), we calculated ${LW}_{mea}$ (Fig. 10c). The determination of *C*_*f*_ was carried out using Eq. (7) (Fig. 10d). Within Fig. 10d, the highest *C*_*f*_ value (C_**F**_-max = 0.55) indicated by the black circle in Fig. 10d) was observed at specific parameter values: *K* = 306.56 mV, *z*_*o*_ = 4.8 m, *x*_*o*_ = − 25 m,* q* = 0.5, and *θ* = − 71.65°, as presented in Table 7. The computed errors for the estimated parameters *K*, *z*_*o*_,* x*_*o*_,* q*, and *θ* were found to be 22.62%, 20%, 0%, 0%, and 4.47%, respectively.

**Table 7:** The authentic and retrieved model parameters pertaining to the second theoretical example (self-potential composite anomaly induced by vertical cylinder and first order regional source) contaminated with 15% RGN and 15% WGN.

Model parameters	True	Recovered	
		15% RGN	15% WGN
K (mV)	250	306.56	293.3108
*z*_*o*_ (m)	4	4.8	5.1
*x*_*o*_ (m)	− 25	− 25	− 25
q	0.5	0.5	0.5
θ (º)	− 75	− 71.65	− 71.13
C_F_-max		0.5462	0.5876

**Figure 11:**
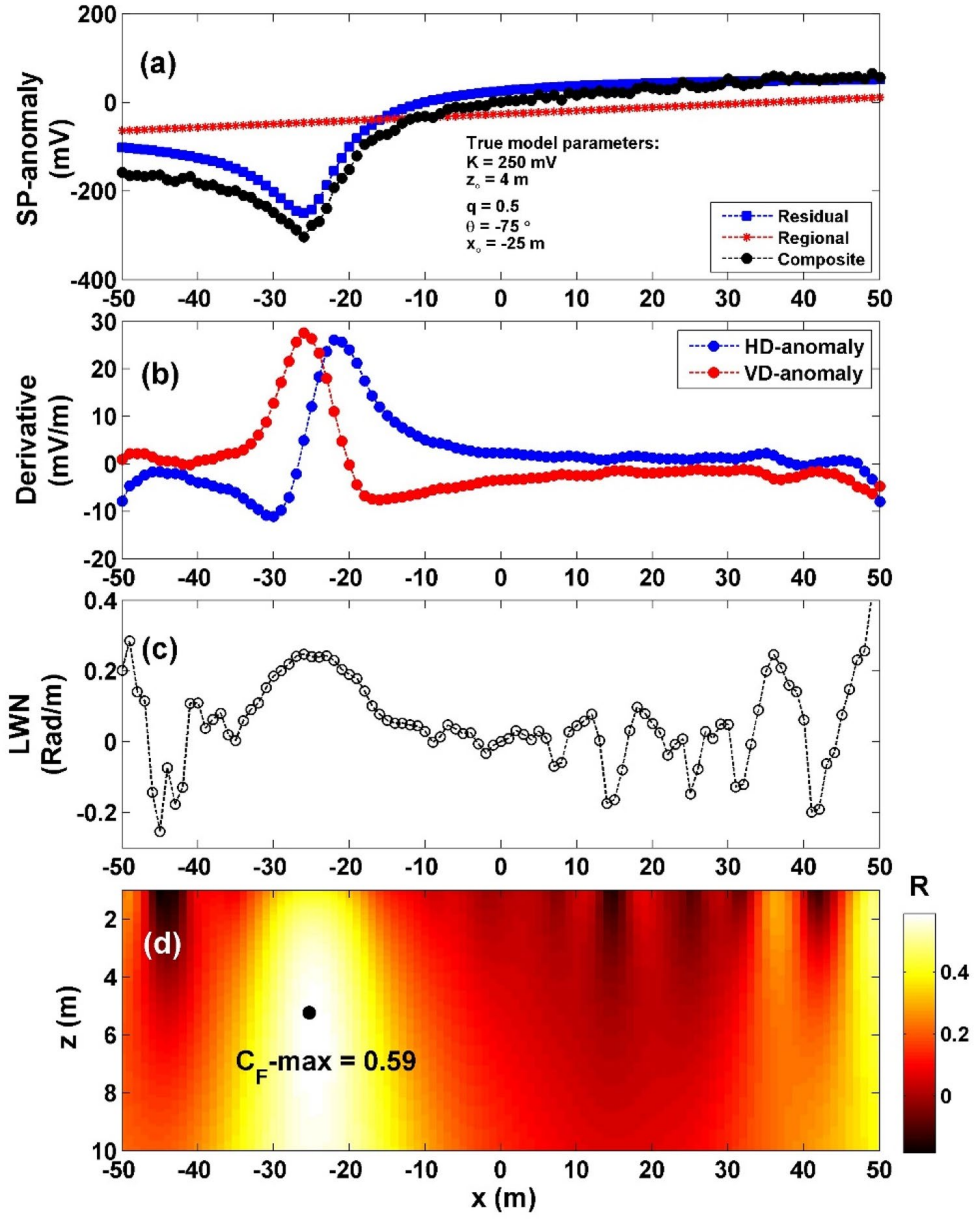
(**a**) Profile of the self-potential anomaly depicted in Fig. 9a after contaminating with 15% WGN, (**b**) computed horizontal and vertical derivatives for the profile depicted in (**a**), (**c**) local wavenumber of the data depicted in (**b**), (**d**) visualizing the correlation factor (*C*_*f*_) and determining the C_**F**_-max through the newly established method.

Moving on to the scenario with 15% WGN (illustrated in Fig. 11a), we again computed the vertical and horizontal gradients of the noisy data (Fig. 11b). Subsequently, employing Eq. (4), we determined ${LW}_{mea}$ (Fig. 11c). To derive *C*_*f*_, Eq. (7) was utilized (Fig. 11d). Within Fig. 11d, the highest *C*_*f*_ value (C_**F**_-max = 0.588), depicted by the black circle in Fig. 11d was observed at specific parameter values: *K* = 293.31 mV, *z*_*o*_ = 5.1 m, *x*_*o*_ = − 25 m,* q* = 0.5, and *θ* = − 71.13°, as indicated in Table 7. The computed errors for the estimated parameters *K*, *z*_*o*_,* x*_*o*_,* q*, and *θ* were found to be 17.32%, 27.5%, 0%, 0%, and 5.16%, respectively.

## Field models

In order to evaluate the effectiveness of the suggested method, it was employed in three distinct real-life field data, including one from India, one from Germany and the third from USA.

### India field example (Neem-Ka-Thana Copper Belt)

The Neem-Ka-Thana Copper Belt in India is distinguished by the prevalence of copper mineralization in the region^4,42^. Notably, the copper deposits are primarily located along fault lines and shear planes, indicating a geological association with these structural features. The concentration of copper in the mines within the Neem-Ka-Thana Copper Belt shows variability, ranging from 0.6 to 1.2%^4,43^. This diversity in copper content underscores the geological complexity of the region, suggesting that mineralization processes have been influenced by a combination of tectonic forces and geological phenomena. Therefore, the Neem-Ka-Thana Copper Belt stands out as a significant geological site where the interplay of geological structures and mineralization processes contributes to the formation of valuable copper deposits.

**Figure 12:**
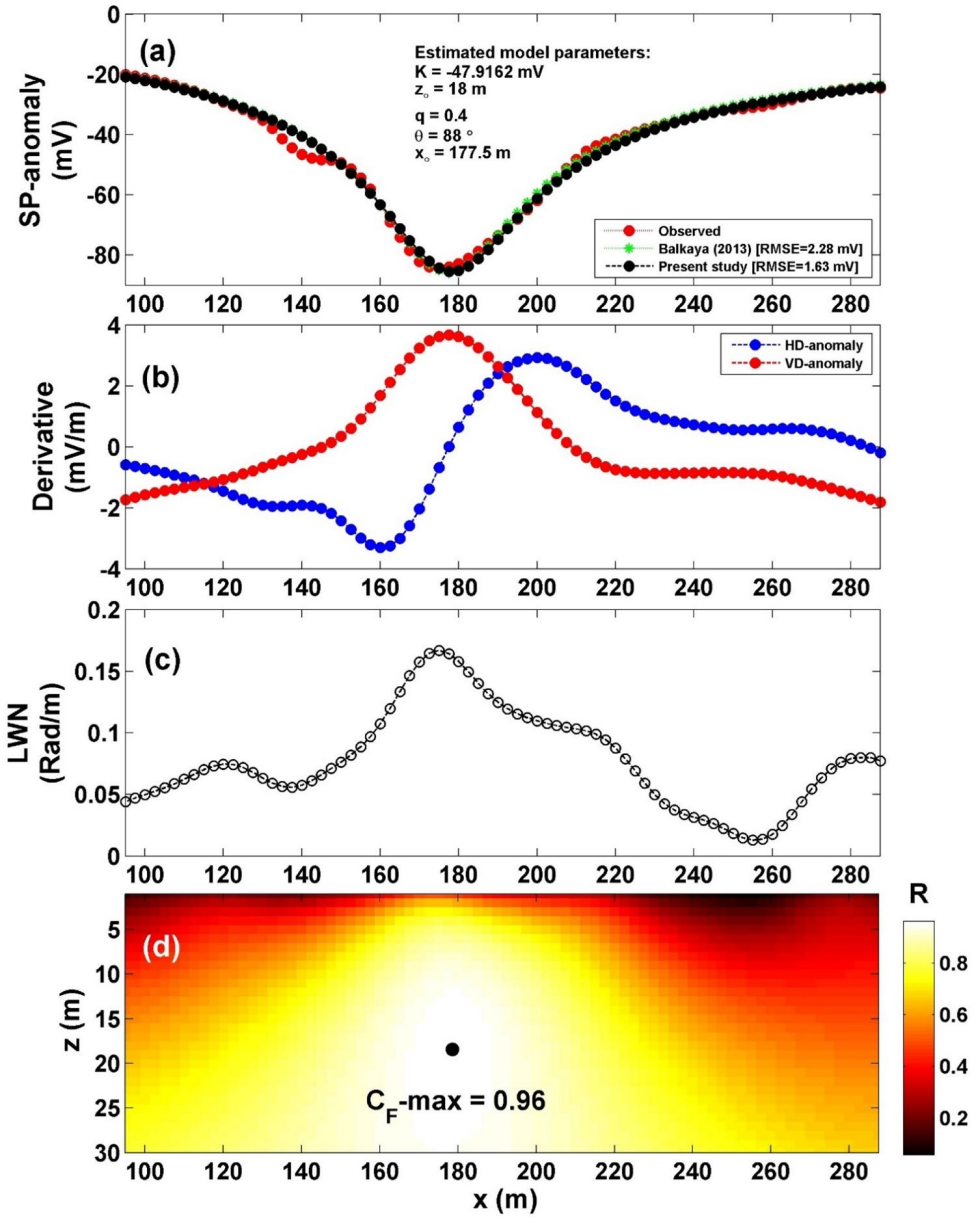
(**a**) Profile of the self-potential anomaly over Neem-Ka-Thana Copper Belt in India, along with the calculated responses from the present study and those obtained by Balkaya^44^. (**b**) Computed horizontal and vertical derivatives for the profile depicted in (**a**), (**c**) local wavenumber of the data depicted in (**b**), (**d**) visualizing the correlation factor (*C*_*f*_) and determining the C_**F**_-max through the newly established method.

A profile of self-potential was acquired over the Neem-Ka-Thana Copper Belt in India^4,31,43^ (Fig. 12a). The profile length was 285 m long. The interpretation procedure initiated with the computation of both the horizontal and vertical gradients of the observed anomaly, illustrated in Fig. 12b. Following this, Eq. (4) was applied to determine the value of ${LW}_{mea}$, as shown in Fig. 12c. Progressing further, Eq. (7) was employed to compute *C*_*f*_, depicted in Fig. 12d, considering various *q* values as presented in Table 8. It is noteworthy that Table 8 presents the maximum value of *C*_*f*_ (C_F_-max = 0.96), represented by a black circle in Fig. 12d, corresponding to *K* = − 47.92 mV, *z*_*o*_ = 18 m, *x*_*o*_ = 177.5 m,* q* = 0.4, and *θ* = 88° (Table 9). The comparison between the results obtained by our suggested method and those obtained by Balkaya^44^ is depicted in Fig. 12a. Table 9 displays a comparison of the inverted parameters between the proposed method and those of different methods found in the literature.

**Table 8:** The correlation factor (***C***_***f***_) calculated at the different shape factors for the Neem-Ka-Thana Copper Belt field example in India.

Geometric shape factor	Maximum correlation factor
**(q)**	**(C**_**F**_**-max)**
**0.5**	**0.9630**
1	0.9628
1.5	0.9579

The optimum values are in [bold].

**Table 9:** Retrieved model parameters for the Neem-Ka-Thana Copper Belt field example in India, and the comparison of the inverted results between the proposed method and those of different methods found in the literature.

Model parameters	Balkaya^44^	Göktürkler and Balkaya^31^			Biswas^4^	Sungkono^43^		Present study
		GA	PSO	SA	(2017)	MDE	µJADE	
K (mV)	− 48.50	− 53.99	− 49.53	− 44.62	32.2	− 48.38	− 49.93	− 47.92
*z*_*o*_ (m)	17.3	18.6	17.6	16.34	10.8	18.81	17.91	18.00
*x*_*o*_ (m)	176.8	176.84	176.77	176.92	177.8	178.32	176.66	177.5
q	0.4	0.42	0.4	0.38	0.5	0.41	0.41	0.40
θ (º)	88.05	87.83	88	88.25	89.6	88.95	88.06	88.00

### Germany field example (Lias-epsilon black shales)

The Lias-epsilon black shales in Germany are situated atop a coal maturity high on the Bramsche Massif in Northwest Germany, as described by^45^. The thermal evolution of this region is attributed to the inversion of the Lower Saxony Basin, occurring during the Early Late Cretaceous period, likely in conjunction with mafic intrusions from the Bramsche, Vlotho, and Uchte Massifs at depths of approximately 5–10 km^46–48^.

The heightened thermal maturity of organic materials in the Lias-epsilon black shales is commonly associated with the intrusion of the Bramsche Massif into the Earth's crust. Notably, strong gravity and magnetic anomalies, along with increased coal maturity in the Westphal D coals found in areas such as Ibbenbüren's mining region, are linked to the presence of the Bramsche Massif^49–52^. The thermal heating of the stratigraphic series likely commenced before the Alp era and extends beyond Mesozoic black shales (2–5% C-org) of the Lower Toarcium and Lias-epsilon, as indicated by Mann^53^. The contemporary morphology of the region has been significantly influenced by Pliocene tectonism and Quaternary sedimentation, as highlighted by studies such as those conducted by^54,55^.

**Figure 13:**
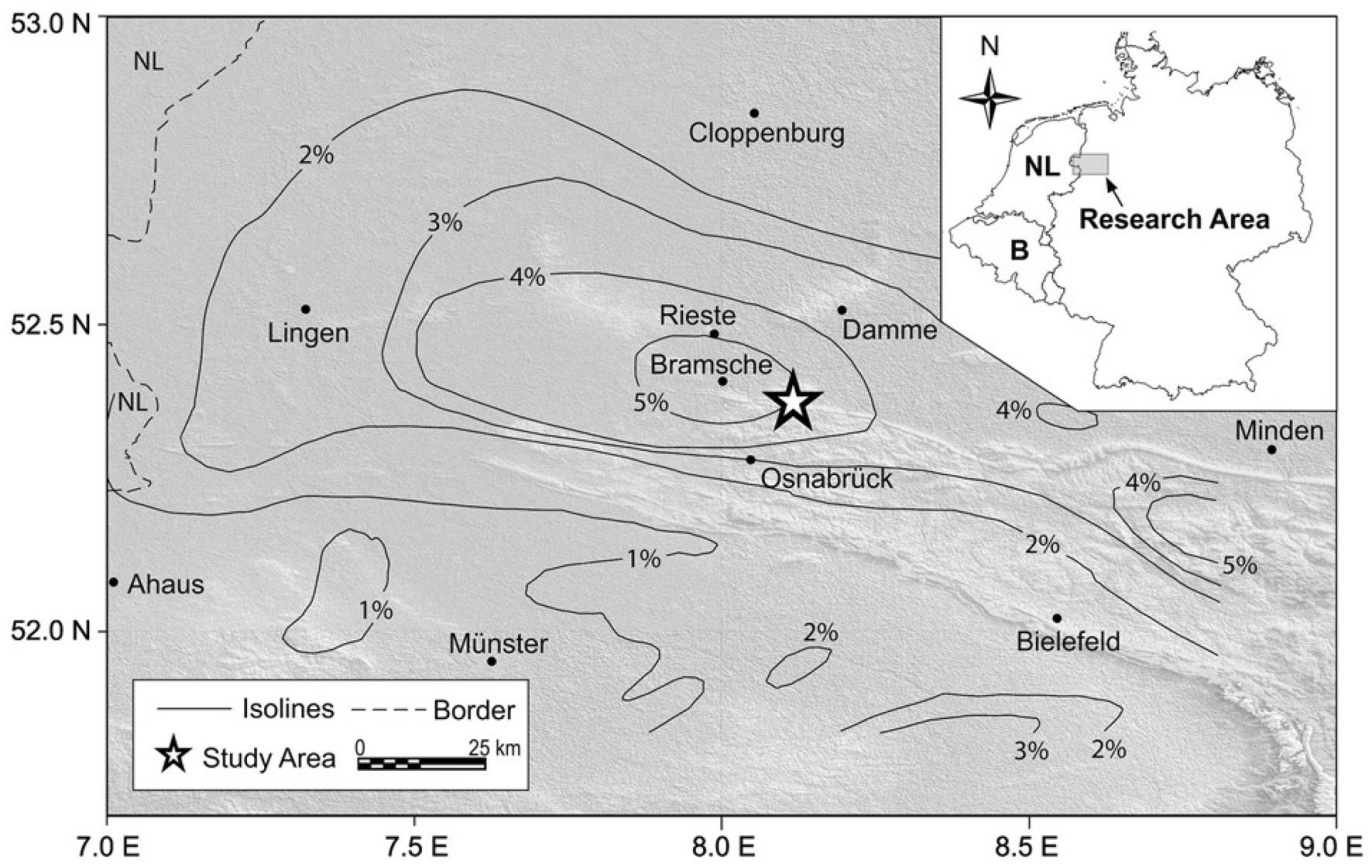
Location map of the of the survey area for the Osnabrück in Germany (after Stadler and Teichmuller^49^ and Gurk et al.^45^). *NL* Netherlands, *B* Belgium.

**Figure 14:**
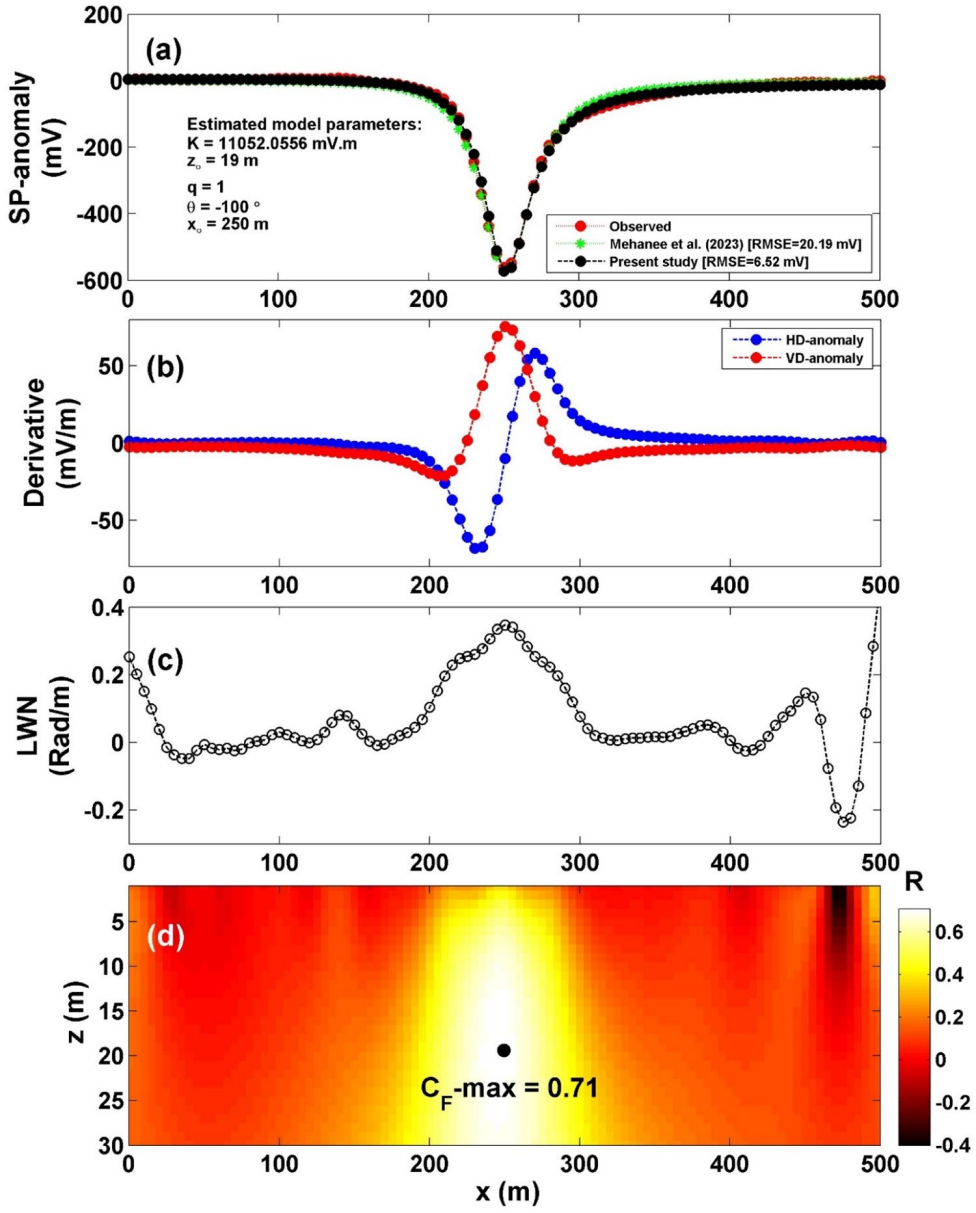
(**a**) Profile of the self-potential anomaly over Lias-epsilon black shales in Germany, along with the calculated responses from the present study and those obtained by Mehanee et al.^57^. (**b**) Computed horizontal and vertical derivatives for the profile depicted in (**a**), (**c**) local wavenumber of the data depicted in (**b**), (**d**) visualizing the correlation factor (*C*_*f*_) and determining the C_**F**_-max through the newly established method.

**Figure 15:**
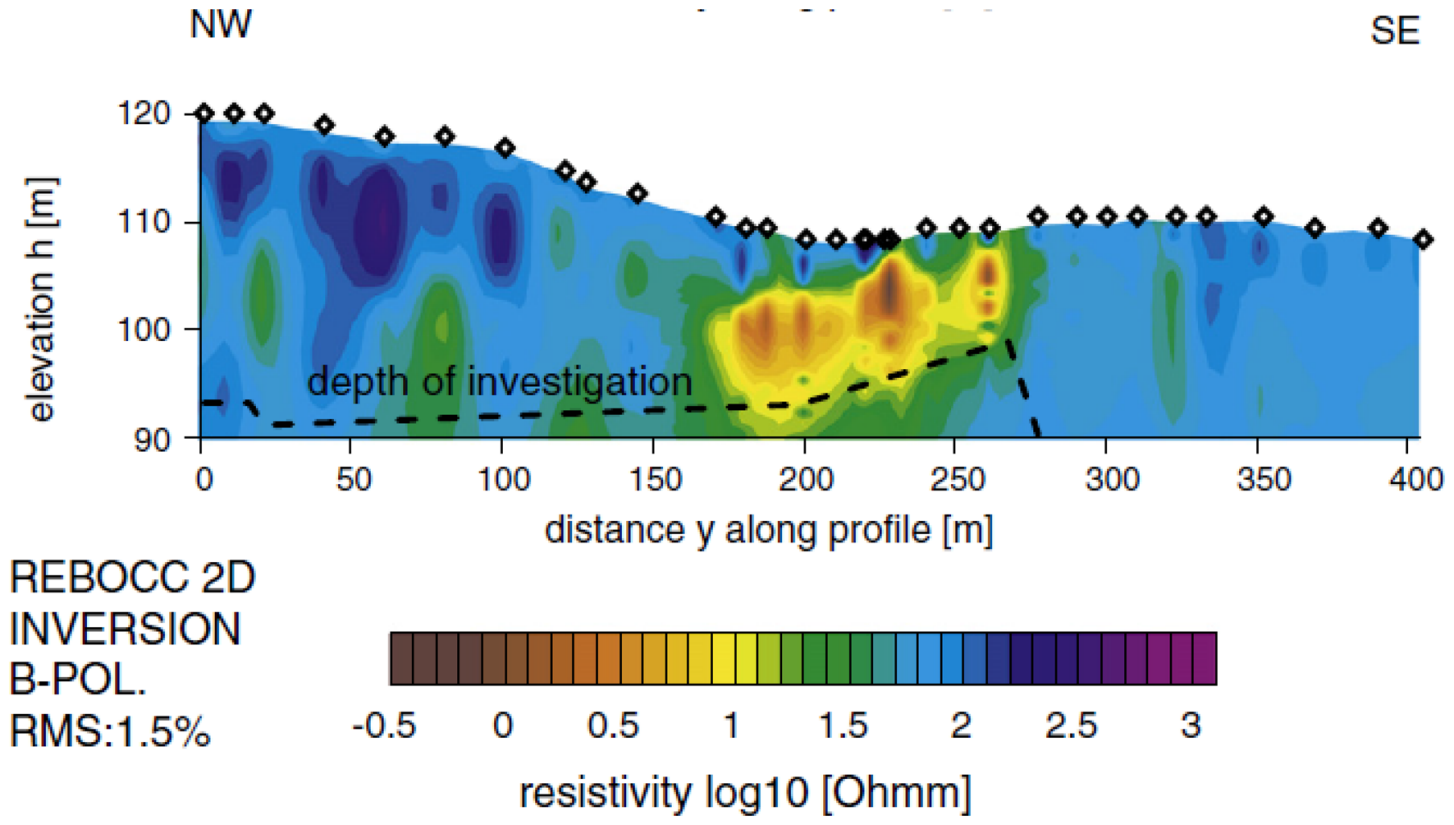
Results of the 2D electrical resistivity tomography inversion cross-section for the Osnabrück anomaly in Germany. (modified from Gurk et al.^45^).

**Figure 16:**
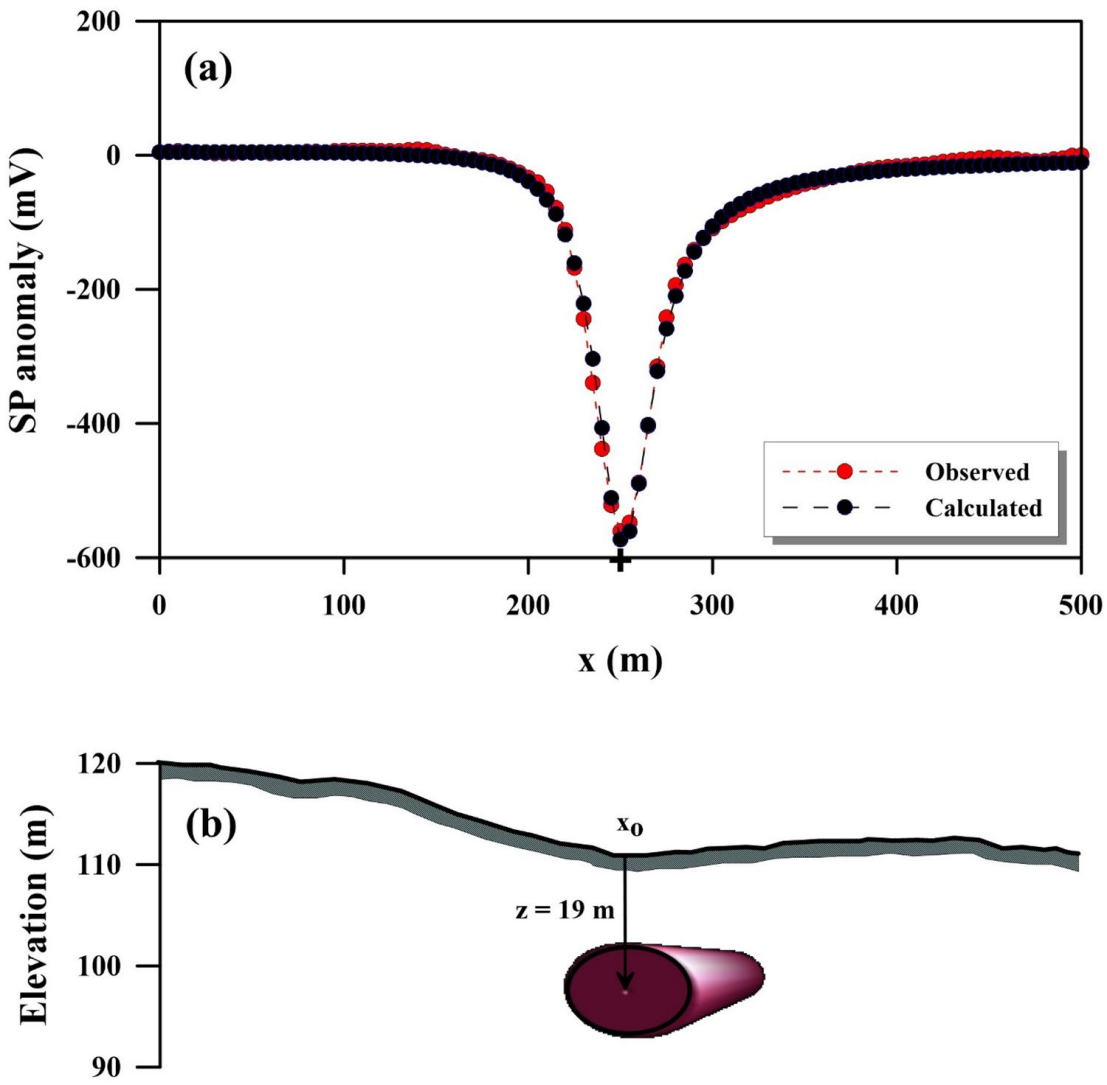
Estimated subsurface model using our suggested technique for the Osnabrück anomaly in Germany, taking into consideration the surface topography (**b**). The plus sign indicates the center location of source anomaly (**a**).

The survey area's location is depicted in Fig. 13^45,49^. A self-potential profile was carried out across a 500 m span over the conductivity anomaly, specifically the Lias-epsilon black shales^45,56^ (Fig. 14a). The interpretation process began by calculating both the horizontal and vertical gradients of the observed anomaly, as illustrated in Fig. 14b. Subsequently, Eq. (4) was utilized to determine the value of ${LW}_{mea}$, as depicted in Fig. 14c. Advancing further, Eq. (7) was applied to calculate *C*_*f*_, shown in Fig. 14d, with consideration for various *q* values outlined in Table 10. It is important to note that Table 10 highlights the maximum value of *C*_*f*_ (C_F_-max = 0.71), denoted by a black circle in Fig. 14d. This corresponds to *K* = 11,052.05 mV m, *z*_*o*_ = 19 m, *x*_*o*_ = 250 m,* q* = 1, and *θ* = − 100° (refer to Table 11). The comparison between the results obtained by our suggested approach and those obtained by Mehanee et al.^57^ is depicted in Fig. 14a. Table 11 provides a comparison of the inverted parameters between the proposed method and those from different methods documented in the literature. Also, Figs. 15 and 16 show the depth estimation from the 2D electrical resistivity tomography and the estimated source model using the suggested technique, respectively (taking into consideration the topography of the area). The results from the 2D electrical resistivity tomography and our method match well.

**Table 10:** The correlation factor (***C***_***f***_) calculated at the different shape factors for the Lias-epsilon black shales field example in Germany.

Geometric shape factor	Maximum correlation factor
**(q)**	**(C**_**F**_**-max)**
0.5	0.6033
**1**	**0.7089**
1.5	0.7084

The Optimum values are in [bold].

**Table 11:** Retrieved model parameters for Lias-epsilon black shales field example in Germany, and the comparison of the inverted results between the proposed method and those of different methods found in the literature.

Model parameters	Gurk et al.^45^	Mehanee^61^	Mehanee et al.^57^	Present study
K (mV m^2q-1^)	− 48.50 (mV)	11,783.60 (mV m)	46,527 (mV m^1.4^)	11,052.05 (mV m)
*z*_*o*_ (m)	10—23	19.9	23	19.00
*x*_*o*_ (m)	251.73	–	250	250.00
q	Thin sheet	1.0	1.2	1.0
θ (º)	95	− 99.2	− 97	− 100.00

### USA field example (Hi'iaka eruption)

On May 5, 1973, a dike penetrated the upper crust of Kilauea volcano within the geologic context of the east rift zone^58^. This intrusion coincided with the eruption of Hi'Iaka and Pauahi craters, as documented by Klein et al.^59^ and Tilling et al.^60^. The dike induced the formation of a surface fissure, stretching 100 m, which erupted magma west-southwest (WSW) of Hi'iaka crater. Geophysical measurements indicated that the dike extended underground in the WSW direction for an additional 1.5 km^58^.

**Figure 17:**
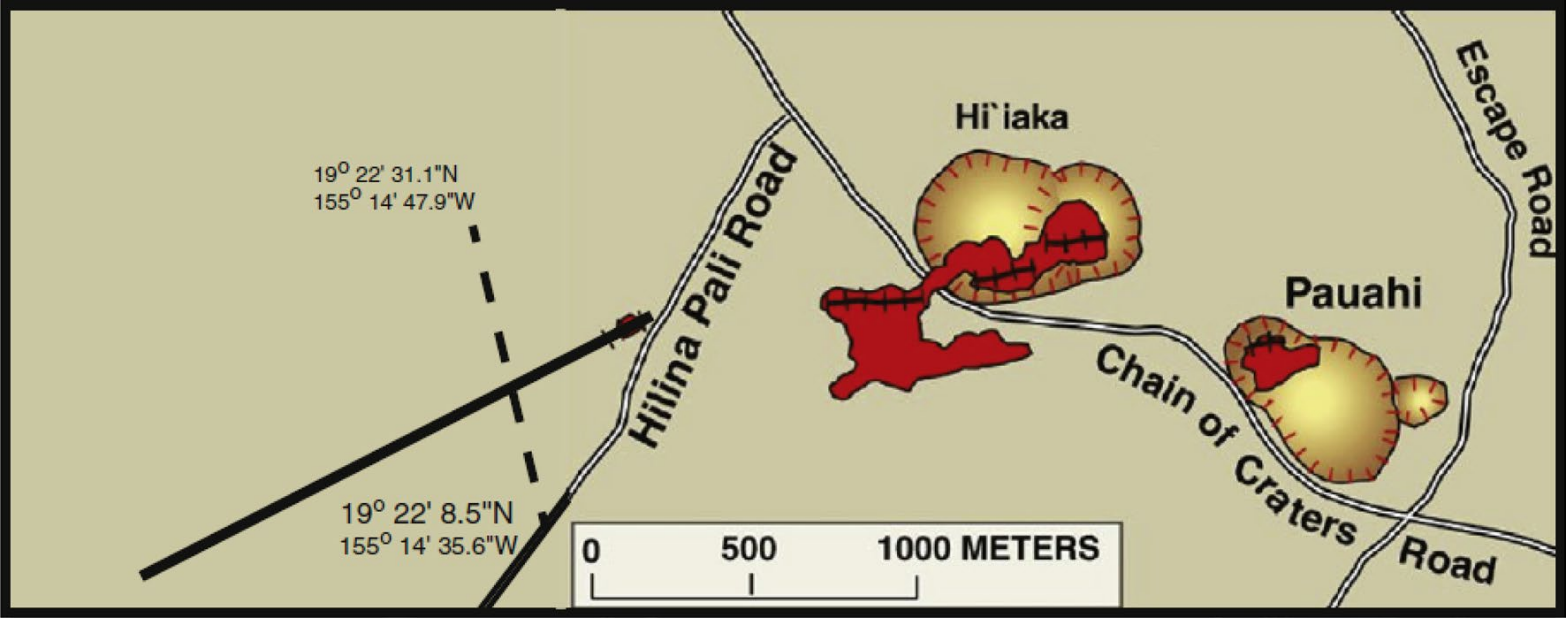
Location map of the of the SP profile for the Hi'iaka eruption in USA (after Davis^58^).

**Figure 18:**
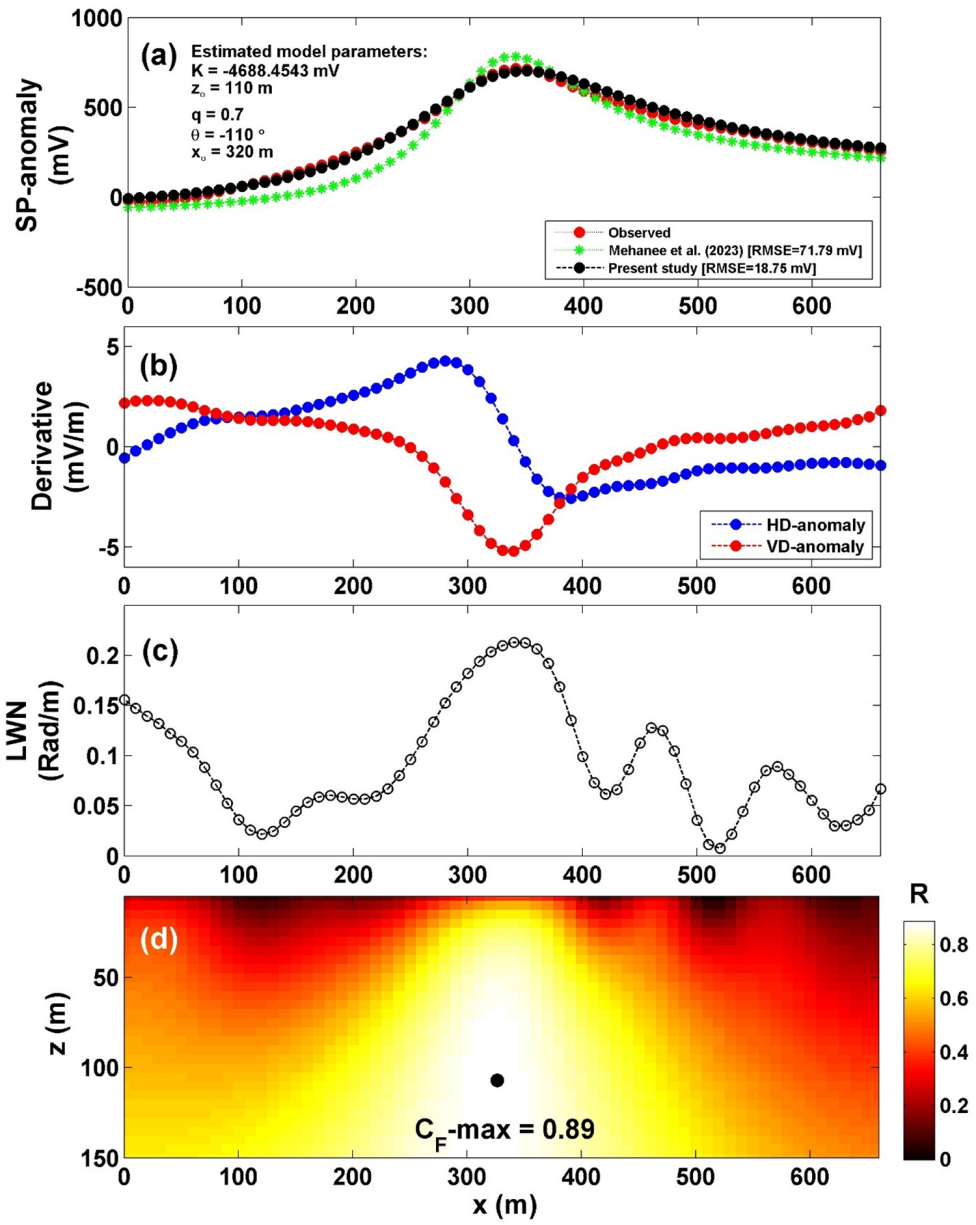
(**a**) Profile of the self-potential anomaly over Hi'iaka eruption in USA, along with the calculated responses from the present study and those obtained by Mehanee et al.^57^. (**b**) Computed horizontal and vertical derivatives for the profile depicted in (**a**), (**c**) local wavenumber of the data depicted in (**b**), (**d**) visualizing the correlation factor (*C*_*f*_) and determining the C_**F**_-max through the newly established method.

The SP profile's location is depicted in Fig. 17^58^. The selected profile was carried out in 1997^58^, spanning a length of 650 m (see Fig. 18a). The process of interpretation commenced by computing both the horizontal and vertical gradients of the observed anomaly, as depicted in Fig. 18b. Subsequently, Eq. (4) was applied to ascertain the value of ${LW}_{mea}$, as illustrated in Fig. 18c. Progressing further, Eq. (7) was employed to compute *C*_*f*_, as shown in Fig. 18d, considering various *q* values outlined in Table 12. It is noteworthy that Table 12 highlights the maximum value of *C*_*f*_ (C_F_-max = 0.89), represented by a black circle in Fig. 17d. This corresponds to *K* = − 4688.45 mV m^2q-1^, *z*_*o*_ = 110 m, *x*_*o*_ = 320 m,* q* = 0.7, and *θ* = − 110° (refer to Table 13). The comparison between the results obtained by our suggested method and those obtained by Mehane et al.^57^ is depicted in Fig. 18a. Table 13 presents a comparison of the inverted parameters between the proposed method and those documented in the literature from different methods.

**Table 12:** The correlation factor (***C***_***f***_) calculated at the different shape factors for the Hi'iaka eruption field example in USA.

Geometric shape factor	Maximum correlation factor
**(q)**	**(C**_**F**_**-max)**
**0.5**	**0.8902**
1	0.8876
1.5	0.8858

The Optimum values are in [bold].

**Table 13:** Retrieved model parameters for the Hi'iaka eruption field example in USA, and the comparison of the inverted results between the proposed method and those of different methods found in the literature.

Model parameters	Davis^58^	Mehanee et al. ^57^	Present study
K (mV m^2q-1^)	–	− 4340	− 4688.45
*z*_*o*_ (m)	120	69	110.00
*x*_*o*_ (m)	–	310	320.00
q	Dike model	0.7	0.70
θ (º)	–	− 110	− 110.00

## Conclusions

In this study, we implemented an effective inversion imaging algorithm to characterize self-potential data originating from diverse sources such as spheres, vertical cylinders, and horizontal cylinders. The demonstrated algorithm holds promise for applications in mineral, ore exploration, and geothermal investigation offering precise predictions of various structural parameters—namely, amplitude factor (*K*), depth (*z*_*o*_), body origin (*x*_*o*_), shape factor (*q*), and polarization angle ($\theta )$—with high accuracy and without the need for a priori information. The suggested algorithm employs the correlation factor (*C*_*f*_) between the local wavenumber of the observed self-potential field and that of the computed field. The findings indicate that the maximum *C*_*f*_ (C_**F**_-max) corresponds to the most reliable estimated model. Moreover, our proposed approach presents an imaging algorithm that provides rapid (within seconds) and robust imaging for subsurface depth and the location of concealed anomalous sources. To validate the efficiency, accuracy, and stability of the proposed algorithm, we subjected it to testing using three synthetic cases, including a pure data, a noisy data contaminated with different types of noise (RGN and WGN), an example for multi-source model and data with regional background effects. The applicability of the algorithm was further assessed through three real cases for mineral/ore exploration and geothermal investigation in India, Germany and USA. The resulting models from these real cases exhibited strong correlations with drilling data and findings reported in the literature. Finally, our study supports the suitability of the proposed algorithm for mineral/ore deposits exploration and geothermal investigation as well.

## Data Availability

The datasets used and/or analyzed during the current study available from the corresponding author on reasonable request.
